# Plasma Cytokine Profiles in Subjects with High-Functioning Autism Spectrum Disorders

**DOI:** 10.1371/journal.pone.0020470

**Published:** 2011-05-27

**Authors:** Katsuaki Suzuki, Hideo Matsuzaki, Keiko Iwata, Yosuke Kameno, Chie Shimmura, Satomi Kawai, Yujiro Yoshihara, Tomoyasu Wakuda, Kiyokazu Takebayashi, Shu Takagai, Kaori Matsumoto, Kenji J. Tsuchiya, Yasuhide Iwata, Kazuhiko Nakamura, Masatsugu Tsujii, Toshirou Sugiyama, Norio Mori

**Affiliations:** 1 Research Center for Child Mental Development, Hamamatsu University School of Medicine, Hamamatsu, Japan; 2 Department of Psychiatry and Neurology, Hamamatsu University School of Medicine, Hamamatsu, Japan; 3 Department of Psychiatry, National Hospital Organization Tenryu Hospital, Hamamatsu, Japan; 4 Department of Psychiatry, Koujin Hospital, Nagoya, Japan; 5 Faculty of Sociology, Chukyo University, Toyota, Japan; 6 Department of Child and Adolescent Psychiatry, Hamamatsu University School of Medicine, Hamamatsu, Japan; Rikagaku Kenkyūsho Brain Science Institute, Japan

## Abstract

**Background:**

Accumulating evidence suggests that dysregulation of the immune system is involved in the pathophysiology of autism spectrum disorders (ASD). The aim of the study was to explore immunological markers in peripheral plasma samples from non-medicated subjects with high-functioning ASD.

**Methodology/Principal Findings:**

A multiplex assay for cytokines and chemokines was applied to plasma samples from male subjects with high-functioning ASD (n = 28) and matched controls (n = 28). Among a total of 48 analytes examined, the plasma concentrations of IL-1β, IL-1RA, IL-5, IL-8, IL-12(p70), IL-13, IL-17 and GRO-α were significantly higher in subjects with ASD compared with the corresponding values of matched controls after correction for multiple comparisons.

**Conclusion/Significance:**

The results suggest that abnormal immune responses as assessed by multiplex analysis of cytokines may serve as one of the biological *trait* markers for ASD.

## Introduction

Autism spectrum disorders (ASD) are a group of neurodevelopmental disorders characterized by pervasive abnormalities in social interaction and communication, and repetitive and restricted behavioral patterns and interests. ASD include autistic disorder, Asperger's disorder and pervasive developmental disorder, not otherwise specified [1]. Susceptibility to ASD is clearly attributable to genetic factors [2], but the etiology of ASD is unknown, and no biomarkers have yet been proven to be characteristic of ASD.

Accumulating evidence suggests that dysregulation of the immune system may be implicated in the pathophysiology of ASD [3], [4]. For instance, postmortem studies have shown that the protein levels of tumor necrosis factor α (TNF-α) and interleukin (IL)-6 [5], as well as the number of activated microglia [6], are significantly increased in the brains of subjects with ASD compared to controls. In addition, lipopolysaccharide-stimulated productions of TNF-α and IL-6 have been shown to be greater in peripheral blood mononuclear cells from subjects with ASD than those from controls [7]. And increased levels of inflammatory cytokines have been detected even in peripheral samples such as serum [8]–[13] or plasma [14]–[18] of patients with ASD. These findings suggest that the pattern of plasma cytokine levels could serve as a useful biological marker of ASD. However, the results of the previous studies addressing serum or plasma levels of cytokines in ASD appear to be inconsistent, probably due to variations in the experimental designs, diagnostic criteria used and age ranges of the subjects, although another possible explanation is that these inconsistencies reflect the heterogeneity of the ASD themselves.

Recent advances in multiplex technologies have enabled measurement of multiple analytes simultaneously. Multiplexing provides data on a large number of analytes, even when the sample volumes are limited [19], [20]. In this study, we used a multiplex assay to measure a series of 48 cytokines in plasma samples from subjects with high-functioning ASD in comparison with matched control subjects. A recent systemic serum proteome profiling study reported that males and females with Asperger's disorder have distinct biomarker fingerprints [11]. Therefore, to prevent any potential confounding effect of sex, we recruited only males in this study. Also, cytokine profiles were only determined in the ASD male subjects who were more than 6 years of age, because a multiplex analysis of cytokines in plasma samples obtained from children less than 5 years of age (the majority of whom were males) was recently reported [14].

## Results

### Subjects

The characteristics of all participants are summarized in Table 1. There was no significant difference in the distribution of age (t = 0.26, P = 0.79) or full IQ (t = 0.46, P = 0.65) between the two groups, indicating that the subject matching was successful. Several pro-inflammatory cytokines, including TNF-α and IL-6, are known to be produced by adipose tissue, and the plasma levels of these cytokines have been correlated with parameters of obesity [21]. Therefore, we measured the weight and height of all the participants, and the body mass index (BMI) was calculated. There were no significant inter-group differences in the weight, height, or BMI. In subjects with ASD, 21 subjects with ASD were diagnosed with autistic disorder and the remaining 7 were considered to have PDD-NOS, according to the Autism Diagnostic Interview-Revised (ADI-R) [22].

**Table 1:** Demographic and clinical characteristics.

Characteristic	Mean (SD) [Range]	
	Control, *N* = 28	ASD, *N* = 28
Age, years	12.3 (2.3) [7]–[15]	12.1 (3.3) [7]–[15]
Full IQ	101.5 (11.5) [82–124]	99.6 (18.6) [72–136]
Height, cm	149.6 (12.5) [121.4–172.8]	147.1 (17.0) [110.0–175.0]
Weight, kg	40.4 (10.6) [24.0–62.0]	40.4 (13.2) [17.5–72.4]
BMI, kg/m^2^	17.7 (2.4) [14.4–25.3]	18.1 (2.6) [13.9–24.2]
Scores on Autism Diagnostic Interview-Revised		
Domain A (social)	N/A	19.9 (5.2) [9]–[27]
Domain BV (communication)	N/A	13.7 (4.0) [8]–[21]
Domain C (stereotype)	N/A	5.6 (2.2) [3]–[10]
Domain D (age of onset)	N/A	3.2 (1.1) [1]–[5]

Abbreviations: ASD, autism spectrum disorder; IQ, intelligence quotient; BMI, body-mass index; and N/A, not applicable.

### Plasma levels of cytokines and chemokines by multiplex assay kits

**Figure 1:**
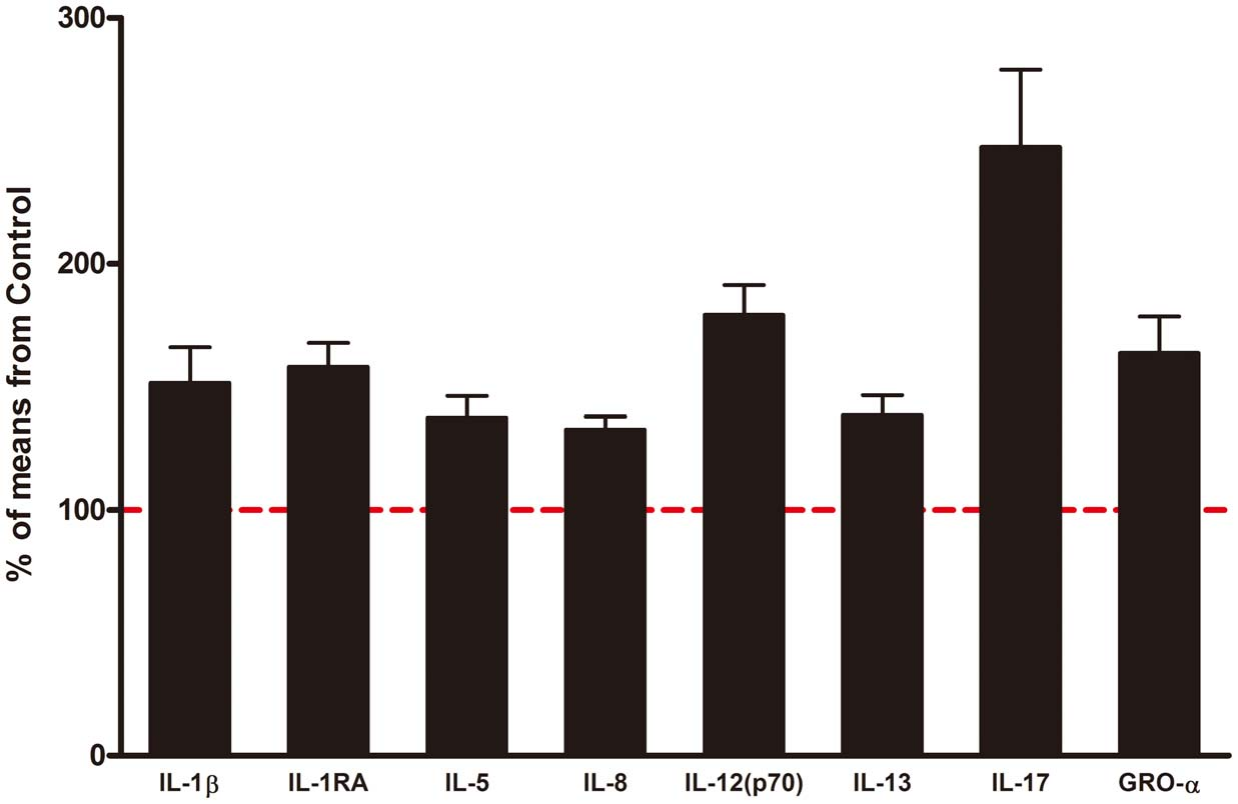
Fold changes of analytes measured by multiplex assay kits in subjects with autism spectrum disorder. The results represent the % concentration relative to the mean concentration of each analyte in the control group (dashed line in red). Data are expressed as the mean plus standard error of the mean.

The comparison of cytokine and chemokine detection is summarized in Table 2. Among a total of 48 analytes, plasma concentrations of IL-2, IL-15, basic FGF, GM-CSF and LIF did not reach the detection range in either group, and these five analytes were excluded from further analyses. Plasma levels of IL-1β, IL-1RA, IL-5, IL-8, IL-12(p70), IL-13, IL-17 and GRO-α were significantly higher in subjects with ASD compared with the corresponding values of matched controls after correction for multiple comparisons. Plasma levels of IL-4, IL-7, G-CSF, IFN-γ, MIP-1β, PDGF-BB, TNF-α, HGF and VEGF tended to be greater in the ASD group than in the control groups, but after correction for multiple comparisons, the differences did not reach the level of statistical significance. The mean levels of fold changes of the cytokines that differed significantly between the two groups are summarized in Figure 1.

**Table 2:** List of analytes in the multiplex assay.

	Control group (n = 28)		ASD group (n = 28)			FDR- corrected *P* value
Analytes	mean	SD	mean	SD	*t* value	
Group I						
IL-1β	1.1	0.8	1.7	0.8	−2.616	*0.049
IL-1RA	85.4	49.2	135.0	43.4	−4.002	*0.003
IL-2	BDR		BDR		-	-
IL-4	2.1	0.9	2.7	0.9	−2.487	0.06
IL-5	2.8	1.4	3.8	1.3	−2.906	*0.033
IL-6	5.9	3.2	6.8	2.4	−1.207	0.37
IL-7	10.8	3.1	12.8	3.4	−2.201	0.09
IL-8	8.7	3.7	11.6	2.5	−3.391	*0.014
IL-9	13.0	10.1	14.8	9.9	−0.672	0.68
IL-10	2.5	1.8	2.9	1.4	−1.039	0.45
IL-12 (p70)	21.3	12.6	38.1	13.7	−4.784	*0.001
IL-13	11.8	5.2	16.3	5.0	−3.329	*0.011
IL-15	BDR		BDR		-	-
IL-17	7.2	4.8	17.7	11.9	−4.287	*0.002
Eotaxin	86.7	50.8	107.6	35.3	−1.789	0.16
Basic FGF	BDR		BDR		-	-
G-CSF	4.8	3.4	6.9	3.2	−2.368	0.07
GM-CSF	BDR		BDR		-	-
IFN-γ	80.0	46.4	107.2	49.6	−2.123	0.10
IP-10	1912.2	3202.5	1075.1	322.0	1.376	0.33
MCP-1	26.4	17.3	28.2	13.9	−0.419	0.83
MIP-1α	6.5	2.6	6.7	2.8	−0.227	0.91
MIP-1β	125.0	45.3	159.8	57.1	−2.527	0.06
PDGF-BB	11053.2	3023.3	12465.3	1548.4	−2.200	0.09
RANTES	6303.5	809.6	6103.5	598.4	1.051	0.46
TNF-α	8.6	9.1	18.0	19.6	−2.316	0.08
VEGF	74.3	65.6	124.9	75.4	−2.682	0.05
Group II						
CTACK	555.8	138.7	606.1	145.8	−1.324	0.33
GRO-α	60.5	38.3	99.0	47.4	−3.347	*0.013
HGF	213.1	83.5	266.0	66.9	−2.619	0.06
IFN-α2	38.4	11.1	38.1	8.2	0.150	0.92
IL-1α	0.5	0.4	0.6	0.4	−0.990	0.47
IL-2Rα	59.6	20.0	56.3	22.2	0.579	0.76
IL-3	17.1	16.6	17.3	9.9	−0.051	0.96
IL-12 (p40)	43.4	23.2	56.2	26.8	−1.907	0.15
IL-16	210.8	90.0	220.6	73.1	−0.447	0.83
IL-18	60.3	24.3	61.3	17.2	−0.171	0.93
LIF	BDR		BDR		-	-
MCP-3	7.2	3.4	5.8	3.7	1.443	0.30
M-CSF	10.7	7.2	13.2	7.6	−1.259	0.35
MIF	78.3	31.3	81.0	28.1	−0.333	0.88
MIG	415.2	270.8	471.3	520.5	−0.505	0.80
β-NGF	3.1	1.9	3.1	1.0	0.059	0.98
SCF	144.0	24.3	150.3	39.3	−0.718	0.68
SCGF-β	29762.4	6331.6	32684.0	5613.8	−1.827	0.16
SDF-1α	162.7	55.7	180.8	43.6	−1.347	0.33
TNF-β	3.4	4.6	3.1	3.4	0.285	0.88
TRAIL	160.7	58.9	133.9	48.9	1.851	0.16

Concentrations of analytes are shown in [pg/ml]. Note the statistically significant difference between the two groups (*P<0.05 after FDR correction for multiple comparisons). Abbreviations: ASD, autism spectrum disorder; BDR, below the detection range; FDR, false discovery rate; and SD, standard deviation.

We then examined the correlations between plasma levels of IL-1β, IL-1RA, IL-5, IL-12(p70), IL-13, IL-17 and GRO-α and clinical variables in the subjects with ASD. There were no statistically significant correlations between the plasma levels of analytes and clinical variables, including age, weight, height, BMI, IQ (full, verbal and performance) and severities in autistic symptoms as assessed by the ADI-R. When correlation coefficients were evaluated among the 7 analytes that showed significant elevation in ASD, there were significant correlations between IL-1β and IL-1RA (Pearson's *r* = .626, *P*<0.001), between IL-5 and IL-13 (*r* = .497, *P* = 0.007), between IL-13 and IL-12(p70) (*r* = .747, *P*<0.001) and between IL-8 and GRO-α (*r* = .415, *P* = 0.028).

## Discussion

In the present study, plasma levels of IL-1β, IL-1RA, IL-5, IL-8, IL-12(p70), IL-13, IL-17 and GRO-α in the high-functioning male subjects with ASD were significantly higher than those of carefully matched control subjects. Our participants with ASD showed no signs or symptoms implying inflammatory diseases and were similar in parameters of obesity, including BMI, to controls. Thus, it is likely that the elevations in plasma levels of those analytes were significantly associated with the diagnosis of ASD. These results are in line with the studies mentioned above, which reported altered immune responses in individuals with ASD [3], [4]. The fold changes of each analyte, however, ranged from 1.5 to 2.5, which values were far lower than those of inflammatory or autoimmune diseases. In addition, none of the analyte plasma levels were correlated with the severity of autistic symptoms. Therefore, it was suggested that the elevation of cytokines observed here may represent an abnormal steady-state immune response in subjects with ASD, and that such a multiplex analysis of cytokines may serve as one of the biological trait markers for the disorder.

### Plasma levels of IL-1β and IL-1RA were elevated in ASD

IL-1β is a pro-inflammatory cytokine produced by various sources, including monocytes, macrophages, dendritic cells, neutrophil leukocytes and endothelial cells [23]. Among previous reports, two studies that examined serum levels of selected cytokines, i.e., IL-1 [12] and IL-1β [24], in autistic subjects reported no change. However, two other recent studies using multiplex assay in ASD have demonstrated a significant increase in plasma IL-1β levels in 2- to 5-year-old children with ASD [14] or in serum IL-1β levels in adults with Asperger's syndrome [11]. Given the wide variety of functions of IL-1β as an important mediator of inflammatory response, including cell proliferation, differentiation and apoptosis [23], it is not surprising that this cytokine can serve as a marker for abnormal response in subjects with ASD. On the other hand, IL-1RA binds to the cell surface IL-1 receptor, inhibits the activities of IL-1β, and modulates IL-1-related immune responses [23]. In this study, plasma levels of IL-1β in subjects with ASD were significantly and positively correlated with those of IL-1RA, suggesting that IL-1RA might have increased as a negative feedback regulator in response to the elevation of IL-1β levels in ASD.

### Increases in plasma levels of IL-5, IL-13 and IL-12p70 in ASD

IL-5 is mainly produced by T helper 2 (Th2) cells and mast cells, and belongs to the Th2 cytokine family [25]. IL-5 stimulates B cells to secrete immunoglobulins and is also a mediator of eosinophil differentiation and activation. Previous studies have reported eosinophilia in children with autism [26], [27], though a negative result has also been reported [28]. IL-13 is another Th2 cytokine that stimulates B cells to secrete IgE, which is an important mediator of allergic inflammation. A trend toward elevation in plasma levels of IL-4, the major Th2 cytokine, was also observed in the current study (t = −2.49, corrected *P* = 0.06, see Table 2). In contrast, plasma levels of IFN-γ and IL-2, which are Th1 cytokines, were similar between subjects with ASD and controls. Plasma levels of IL-2 and IFN-γ have been reported to be increased in autism (n = 20, mean age = 10.7 years) [17]. However, since none of the other studies found elevations of IL-2 or IFN-γ in peripheral samples [9], [11], [14], [18], it was suggested that elevation of these Th1 cytokines may not be common in subjects with ASD. Our findings of increased levels of Th2 cytokines without corresponding changes in Th1 cytokines were consistent with previous *in vitro* studies which demonstrated Th2-preferred responses after stimulation in peripheral blood monocytes from subjects with ASD [26], [29]. Since Th2 cells have been shown to play a role in the pathogenesis of allergy [30], our current findings are not inconsistent with the fact that allergy is a common clinical problem in individuals with ASD [31], [32].

IL-12(p70) is a heterodimeric cytokine that consists of two subunits, p35 and p40 [33]. Our current finding of elevated plasma levels of IL-12(p70) is consistent with previous results in children with autism [17] and adults with Asperger's syndrome [11]. IL-12(p40) has also been shown to be increased in the plasma of children with ASD [14]. IL-12(p70) is an immunoregulatory cytokine that is produced mainly by B cells and by monocytes, and is involved in the differentiation of naive T cells into Th1 cells [33]. The elevated levels of plasma IL-12 in ASD might be an unsuccessful compensation for the above-mentioned Th1/Th2 imbalance in subjects with ASD.

### Increased plasma levels of IL-17, IL-8 and GRO-α in ASD

Outside the Th1/Th2 cell paradigm, a distinct T helper cell subset that produces IL-17 has recently been discovered, and is known as the Th17 cell subset [34]. Th17 cells are responsive to IL23 and secrete IL-17. Enstrom and his colleagues [15] have reported that plasma IL-17 levels in 2- to 5-year-old children with autism were similar to those in controls, but the IL-23 levels were decreased in the children with autism. Researchers from the same group also examined peripheral blood monocytes from subjects with ASD and found that PHA-stimulated release of IL-23, but not IL-17, was lower in subjects with ASD than in controls [35]. The discrepancy between our study and the previous report by Enstrom et al. [15] presumably reflects the differences in the age of participants and experimental designs. In addition, because the kits we used for multiplex assay did not include IL-23 as an analyte, further studies will be needed to examine the effects of this factor. Interestingly enough, however, recent findings suggest that IL-1β, the plasma levels of which were increased in our subjects with ASD, plays an important role in Th17 cell differentiation and IL-17 secretion [36], [37].

Both IL-8 and GRO-α are chemokines produced by macrophages and other cell types, such as epithelial and endothelial cells. These chemokines have chemotactic activity on neutrophils and play important roles in the innate immune response. Previous studies have examined IL-8 levels in plasma or serum, and found either increases [14] or no change [11], [38] in peripheral IL-8 levels. With regard to GRO-α, there has been no report showing a significant difference as compared to controls. The reason why these chemokines are increased in subjects with ASD is currently unknown. However, IL-17 is known to be a potent mediator of production of IL-8 and GRO-α from epithelial cells [34]. Since IL-8 and GRO-α function as chemotaxins of these chemokines, its elevation in the peripheral circulation suggests an activation of innate immunity. That is, the elevation in plasma IL-8 and GRO-α might have resulted from IL-17 secretion by Th17 cells activated in response to subclinical infections in epithelial or endothelial cells in our subjects with ASD.

### Limitations

There were limitations in the present study. The small sample size renders the data presented here preliminary, and a larger study with more subjects with ASD will be necessary. However, recruitment for the current study was limited to a group of high-functioning subjects with ASD, none of whom were given psychotropic drugs. Therefore, our data are free from possible confounding factors and thus reflect a certain common immunological pathology among people with ASD.

## Materials and Methods

### Subjects

Twenty-eight male subjects with ASD and 28 healthy male controls participated in this study. All the participants were Japanese, born and living in restricted areas of central Japan, including Aichi, Gifu, and Shizuoka prefectures. Based on interviews and available information, including hospital records, diagnoses of ASD were made by an experienced child psychiatrist (TS) based on the DSM-IV-TR criteria [1]. The ADI-R [22] was also conducted by two of the authors (KJT and KM), both of whom have an established reliability of diagnosing autism with the Japanese version of ADI-R. ADI-R is a semi-structured interview conducted with a parent, usually the mother, and is used to confirm the diagnosis and also to evaluate the core symptoms of ASD. The ADI-R domain A score quantifies impairment in social interaction, the domain BV score quantifies impairment in communication, and the domain C score quantifies restricted, repetitive and stereotyped patterns of behavior and interests. The ADI-R domain D corresponds to the age of onset criterion for autistic disorder. We also used the third edition of the Wechsler Intelligence Scale for Children [39] to evaluate the intelligence quotient (IQ) of all the participants. Co-morbid psychiatric illnesses were excluded by means of the Structured Clinical Interview for DSM-IV (SCID) [40]. Participants were excluded from the study if they had any symptoms of inflammation, a diagnosis of fragile X syndrome, epileptic seizures, obsessive-compulsive disorder, affective disorders, IQ of lower than 70, or any additional psychiatric or neurological diagnoses. None of the participants had ever received psychoactive medications before this study. Healthy control subjects were recruited locally by advertisement. All control subjects underwent a comprehensive assessment of their medical history to eliminate individuals with any neurological or other medical disorders. SCID was also conducted to scrutinize any personal or family history of past or present mental illness. None of the comparison subjects initially recruited was found to fulfill any of these exclusion criteria. This study was approved by the ethics committee of the Hamamatsu University School of Medicine. All participants as well as their guardians were given a complete description of the study, and provided written informed consent before enrollment.

### Blood sampling and multiplex assay

Fasting blood samples from all the participants were obtained between 11:00 and noon by venipuncture and collected into EDTA-containing tubes. Immediately after the sampling, samples were centrifuged for 10 min at 4°C, divided into 200-µl of aliquots, and stored at −80°C until use. The mean time interval for preparation of plasma from blood samples was 4.5 min (3 to 6 min). Multiplex kits for measuring cytokines and chemokines were purchased from Bio-Rad (Bio-Plex Pro Human Cytokine Group I [27-plex] and Group II [21-plex] panels; Bio-Rad, Hercules, CA). The kits were used per the manufacturer's instructions. Plasma samples were diluted using the appropriate sample diluents provided in each kit in accordance with the manufacturer's instructions. Concentrations (pg/ml) of different analytes in the plasma samples were determined by using the standard curves generated in the multiplex assays. Each standard curve was generated using eight points of concentrations, and a nonlinear least squares minimization algorithm was used for the curve fitting by the five-parameter logistic equation and to determine the high and low limits of detection. Data points for analytes that were occasionally above or below the detection range were discarded.

### Data analysis

Comparisons of concentrations of analytes between subjects with ASD and controls were made by an unpaired *t*-test after confirming that there were no statistically significant differences in variance as assessed by the F test. A *P* value of less than 0.05 was considered to be statistically significant after adjustment for the false discovery rate (FDR) for multiple comparisons using the Benjamin-Hochberg procedure. Evaluation of relationships between plasma levels of analytes and clinical variables among subjects with autism spectrum disorder was performed with Pearson's *r* correlation coefficient. In the correlation analysis, values of *P*<0.05 were regarded as statistically significant. All statistical analyses were performed using SPSS statistics software (version 17; SPSS K.K., Tokyo, Japan).
